# Identification of a *de novo DYNC1H1* mutation via WES according to published guidelines

**DOI:** 10.1038/srep20423

**Published:** 2016-02-05

**Authors:** Dongxue Ding, Zhao Chen, Kai Li, Zhe Long, Wei Ye, Zhaoli Tang, Kun Xia, Rong Qiu, Beisha Tang, Hong Jiang

**Affiliations:** 1 Department of Neurology, Xiangya Hospital, Central South University, Changsha, Hunan, P.R. China; 2 Key Laboratory of Hunan Province in Neurodegenerative Disorders, Central South University, Changsha, Hunan, P.R. China; 3 State Key Laboratory of Medical Genetics, Central South University, Changsha, Hunan, P.R. China; 4 School of Information Science and Engineering, Central South University, Changsha, China

## Abstract

*De novo* mutations that contribute to rare Mendelian diseases, including neurological disorders, have been recently identified. Whole-exome sequencing (WES) has become a powerful tool for the identification of inherited and *de novo* mutations in Mendelian diseases. Two important guidelines were recently published regarding the investigation of causality of sequence variant in human disease and the interpretation of novel variants identified in human genome sequences. In this study, a family with supposed movement disorders was sequenced via WES (including the proband and her unaffected parents), and a standard investigation and interpretation of the identified variants was performed according to the published guidelines. We identified a novel *de novo* mutation (c.2327C > T, p.P776L) in *DYNC1H1* gene and confirmed that it was the causal variant. The phenotype of the affected twins included delayed motor milestones, *pes cavus*, lower limb weakness and atrophy, and a waddling gait. Electromyographic (EMG) recordings revealed typical signs of chronic denervation. Our study demonstrates the power of WES to discover the *de novo* mutations associated with a neurological disease on the whole exome scale, and guidelines to conduct WES studies and interpret of identified variants are a preferable option for the exploration of the pathogenesis of rare neurological disorders.

Recent advances in sequencing technologies and bioinformatics offer the opportunity to more easily identify the disease-causing mutations in rare Mendelian diseases. However, the contribution of genetic variants to sporadic disease remains largely unknown^1^,^2^. It is difficult for researchers to identify the causal gene in apparently sporadic conditions using traditional sequencing approaches, especially in small families^3^. Whole exome sequencing (WES), which enables researchers to interrogate all known protein-coding genes in a single experiment, facilitates the discovery of causal variants in rare diseases^4^. In addition, MacArthur *et al.*^5^ developed guidelines for the investigation causality of sequence variants causality in human disease. The American College of Medical Genetics and Genomics (ACMG) have also developed guidelines for the interpretation of sequence variants^6^.

Recently, many neurological diseases such as alternating childhood hemiplegia^7^, Kabuki syndrome^8^, and amyotrophic lateral sclerosis (ALS)^9^, have been identified that are caused by *de novo* mutations. In this study, we searched for the disease-causing variants by simultaneously sequencing the exome of the proband and her unaffected parents. In our study design and sequencing data analysis, we utilized the two recently published guidelines to investigate the causality of the sequence variants and to interpret the novel variants that were identified via the human genome sequencing. Consequently, we determined that a novel *de novo* missense mutation (c.2327C > T, p.P776L) in the *DYNC1H1* gene was the disease-causing mutation. Our study demonstrated that WES is a preferable strategy for the detection of *de novo* mutations. The objective investigation and interpretation of sequence variants is an accessible means to molecularly diagnose Mendelian diseases.

## Results

### Clinical features

**Figure 1:**
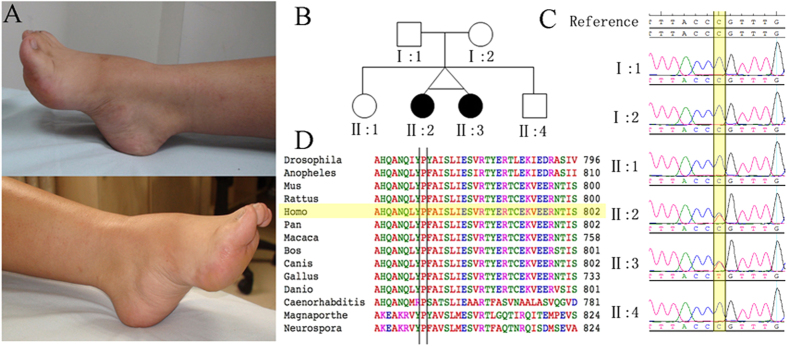
Clinical features of the patients and the pedigree with the c.2327C> T, p.P776L mutation in DYNC1H1. (**A**) Foot deformities (*pes cavus*) of the two patients. (**B**) Pedigree structure of the studied family. In the family, WES was performed in I:1, I:2, and II:3. (**C**) Electropherograms of the Sanger sequences of the *DYNC1H1* c.2327C > T, p.P776L variant; II: 2 and II: 3 are heterozygous mutations. (**D**) The conservation of this variant among different species.

The proband and her twin sister, aged 24 years old, were born from an uneventful pregnancy. The twin sisters had mild foot deformities (*pes cavus*) at birth. Late infantile motor milestones were apparently delayed. The twins could walk independently at approximately 7 years of age but a waddling gait persisted thereafter. Then, the twins slowly developed progressive weakness and wasting of the lower extremity muscles from 9 to 14 years of age. According to the parents, no motor decline was apparent during the last years. A physical examination in our hospital revealed *pes cavus* (Fig. 1A), mild distal lower limb-dominant muscle atrophy and absent deep tendon reflexes. Muscle strength and tendon reflexes were preserved in the upper limbs. No fasciculations or other neurological deficits were observed. No sensory disturbances or ataxia were recognized. The twins demonstrated normal intellectual development according to measurements performed by the Mental Health Center of Xiangya Hospital.

Clinical laboratory evaluations revealed normal blood counts and creatine kinase levels and slightly increased lactic acid levels (3.2 mmol/L before exercise and 3.5 mmol/L after exercise; normal range 1.42–1.90 mmol/L). Non-contrast brain magnetic resonance imaging scans were also normal. Sensory and motor nerve conduction studies revealed normal conduction velocities and amplitudes for the upper and lower extremities. Electromyographic (EMG) recordings revealed a small amount of denervated potentials, which indicated anterior horn involvement in the lower limbs. In addition, large-amplitude and long-duration motor unit potentials were observed in the lower limbs. Neurogenic recruitment patterns were present in all leg muscles. All features were consistent with typical chronic denervation.

### Genetic findings

The average sequencing depth and depth distribution on target of the family are shown in Supplementary Fig. S1. To date, there have been 19 genes (*AARS*, *ATP7A*, *BICD2*, *BSCL2*, *DCTN1*, *DYNC1H1*, *GARS*, *HINT1*, *HSJ1*, *HSPB1*, *HSPB3*, *HSPB8*, *IGHMBP2*, *MYH14*, *PLEKHG5*, *REEP1*, *SLC5A7*, *SETX*, and *TRPV4*) have been associated with lower limb weakness and wasting, and foot deformity^10^,^11^. Using the Integrative Genomics Viewer v.2.3 (IGV, Supplementary Fig. S2), we checked the coverage of these genes manually. No exons or exon-intron regions were located in these genes with <10× coverage.

With the data analysis and variants filtering strategy described above, 5 genes with homozygous variants (*HSD17B7*, *MYH6*, *PCLO*, *DIXDC1*, and *BPTF*), 1 gene with compound heterozygous variants (*MUC19*), and 4 genes (*DYNC1H1*, *PHOX2B*, *AKAP12*, and *NEK5*) with *de novo* variants were identified. Among them *DYNC1H1* is a known causal gene of SMA-LED. Sanger sequencing was conducted to exclude the false positives in the WES. Finally, the *de novo* mutation (c.2327C > T, p.P776L in *DYNC1H1*) in the proband was confirmed by Sanger sequencing (Fig. 1C). This mutation was a true *de novo* mutation as it was absent in her parents. Interestingly, the mutation was present in her twin sister (another patient), but did not exist in her two unaffected siblings (Fig. 1C, Supplementary Table S1). It was not found in more than 60,000 people (including the Exome Aggregation Consortium database, 1000 genomes, dbSNP 141 and the NHLBI Exome Sequencing Project). According to ACMG guidelines, the *de novo* mutation (c.2327C > T, p.P776L in *DYNC1H1*) is categorized to be the disease “pathogenic variant” because it belongs to both PS2 (*de novo* in a patient with the disease and no family history) and PS4 (the prevalence of the variant in affected individuals is significantly increased compared with the prevalence in controls) in ACMG^6^. This variant is located in a highly conserved domain (Fig. 1D) and is “probably damaging” as predicted by PolyPhen2, Mutation Taster, SIFT and CADD (Table 1).

We also conducted Sanger sequencing of the other genes (*HSD17B7*, *MYH6*, *PCLO*, *DIXDC1*, *BPTF*, *MUC19*, *PHOX2B*, *AKAP12*, and *NEK5*). Another variant passed the co-segregation analysis, which was a splicing variant (Table 1; Supplementary Table S1) in *HSD17B7*, with a MAF score 0.0018 in dbSNP. This gene encodes an enzyme that functions both as a 17-beta-hydroxysteroid dehydrogenase (EC 1.1.1.62) during the biosynthesis of sex steroids and as a 3-ketosteroid reductase (EC 1.1.1.270) during the biosynthesis of cholesterol ( http://omim.org/entry/606756). These functions have no overlap with the pathogenesis of SMA-LED. Thus, we suggest that *HSD17B7* is not a candidate gene for our phenotype of interest. The Sanger sequencing results indicated that all the other candidate variants mentioned above did not segregate appropriately in the family or were false positives from WES, and could not be the causative mutations (Supplementary Table S1). Our exome data revealed only one *de novo* mutation, and this result is consistent with previous reports (approximately 1 *de novo* mutation/generation/exome)^12^,^13^.

## Discussion

*DYNC1H1* is located on chromosome 14q32 and encodes the heavy chain of cytoplasmic dynein 1, which is a component of a multi-subunit motor complex that is essential for retrograde axonal transport and other intracellular functions^14^. In humans, *DYNC1H1* mutations are present in autosomal dominant pedigrees of CMT^15^, cHSP (complex hereditary spastic paraplegia)^16^, severe cognitive disability, microcephaly, MCD^17^, and SMA-LED^18^ (Supplementary Table S2). Additionally, *de novo* mutations in *DYNC1H1* are present in patients with sporadic mental retardation and hypotonia^19^, severe intellectual disability and peripheral neuropathy^20^, SMA-LED^21^,^22^, MCD^17^, or a combination phenotype^23^ (Supplementary Table S2).

Here, we suggest the molecular diagnosis to be SMA-LED according to WES, and we determined the causal variant to be a *de novo* SNV (c.2327C > T, p.P776L) located in exon 8 of *DYNC1H1*. The process of our study design, genomic sequence data analysis and the interpretation of the sequencing variants are consistent with the guidelines published by MacArthur *et al.*^5^ and the ACMG^6^. In our study design phase, we identified the disease as a rare Mendelian disorder. The family reported no history of movement disorders. We inferred the mutation type to be a homozygous mutation, a compound heterozygous mutation or a *de novo* mutation. Thus, we utilized WES to sequence the proband and her unaffected parents to explore the potential disease causal variants^5^. Quality controls (QC) were conducted during the whole process, including QC for the DNA library, testing raw sequence data and testing base calling.

After choosing the study strategy and ensuring strict QC during WES, we interpreted the sequence variants in an objective way, as suggested by MacArthur *et al.*^5^ and the ACMG^6^. The ACMG states “investigators should begin by examining sequence variations in genes known to be associated with that phenotype, and assessing sequence coverage of the coding sequences and splice junctions for these genes before exploring the possibility of new candidate genes in the affected individuals”^6^. As mentioned above, 19 genes are currently associated with lower limb weakness and wasting and foot deformity^10^,^11^. However, there were no other known causal genes identified in the WES results except for *DYNC1H1*. *DYNC1H1* is a known causal gene of SMA-LED^18^,^24^,^25^. Then, the question was whether the identified variant was the disease causing mutation or not. The ACMG guideline offers us an objective way to answer this question^6^. Both parental samples were obtained from the biological parents of the patients, and our patient had a family history of disease that was consistent with *de novo* inheritance (e.g., unaffected parents for a dominant disorder). The phenotype (foot deformity at birth, delayed motor milestones and lower limb weakness and atrophy) matched the *DYNC1H1*’s disease association with reasonable specificity. Thus, the *de novo* c.2327C > T, p.P776L mutation in *DYNC1H1* has strong pathogenic criterion (PS2) according to ACMG^6^. Additionally, this mutation was not found in a large control population (including the Exome Aggregation Consortium database, 1000 genomes, dbSNP 141 and the NHLBI Exome Sequencing Project) indicating its strong pathogenic criterion (PS4) according to ACMG^6^. Literature and database sources as well as computational (*in silico*) predictive programs were also considered. Sanger sequencing and co-segregation analyses of all the identified candidate novel variants further confirmed our molecular diagnosis of the patient^5^. We also summarized our results as a supplement (Table 2, Supplementary Section1) according to the ACMG recommendations in order to facilitate the consultation of the proband and her affected twin sister and refer them for prenatal counseling when they become pregnant.

In conclusion, using WES and the related guidelines, we identified a novel *de novo* mutation (c.2327C > T, p.P776L) in the *DYNC1H1* gene and confirmed it as the causal variant of SMA-LED. This paradigm facilitates the molecular diagnosis of rare Mendelian diseases. WES, combined with the guidelines for investigating the causality of sequence variants in human diseases and the interpretation of the sequence variants, as recommended by MacArthur *et al.*^5^ and the ACMG^6^, has become a preferable option to explore the pathogenesis of many rare neurological disorders and to make a molecularly diagnose the diseases.

## Method and Materials

### Subjects

A non-consanguineous Chinese family including healthy parents, two affected twins and two unaffected siblings were enrolled in our study (Fig. 1B). The family presented with no history of movement disorders. Genomic DNA was extracted from peripheral blood leukocytes via standard phenol-chloroform extraction methods. This study was approved by the ethics committee of Xiangya Hospital affiliated to Central South University. The methods in this study were performed in accordance with the approved guidelines. Written informed consent was obtained from all subjects.

### Whole exome sequencing and data analysis

We conducted WES of a proband-parent trio to identify the causal gene. The SureSelect Human All ExonV5 Kit (Agilent) was used for exome capture. The IlluminaHiseq 2500 platform (San Diego, CA) was utilized for genomic DNA sequencing of the proband and her parents in Novogene (Beijing, China).

Raw image analyses and base calling were performed using Illumina’s Pipeline (version 1.3.4) with default parameters. Sequence data were aligned to the reference human genome (hg19) using the Burrows-Wheeler Aligner (BWA)^26^, and duplicate reads were removed using Picard tools. We used the Genome Analysis ToolKit (GATK)^27^ to perform the re-alignment and variation (SNP and Indel) detection. Annovar was utilized to catalogue the detected variations. Then, we filtered variations with a homopolymer length >6 (and synonymous substitutions) or that were common (>0.5%) in dbSNP ( http://www.ncbi.nlm.nih.gov/projects/SNP/), HapMap, and the 1000 Genomes Project ( http://www.1000 genomes.org). Variants that were not present in any of the above databases were considered novel.

Given to the characteristics of the pedigree, homozygous, compound heterozygous or *de novo* variations were considered to be candidate causal variations^28^.

### Polymerase chain reaction (PCR) and Sanger sequencing

We confirmed the candidate causal variations identified via WES and conducted co-segregation analyses among the family. The primers of the candidate variations were designed using Primer 3 ( http://primer3.ut.ee/). The genomic DNA was PCR-amplified using Roche Fast Start PCR Master Mix polymerase (Roche Diagnostics Corp, USA). PCR products were sequenced with Applied BiosystemsBigDye terminator sequencing chemistry and then run on an ABI3730xl genetic analyzer according to the manufacturer’s instructions (Applied Biosystems,CA, USA). Sequence analysis was performed with Lasergene software (DNASTAR, Madison, WI, USA).

## Additional Information

**How to cite this article**: Ding, D. *et al.* Identification of a *de novo DYNC1H1* mutation via WES according to published guidelines. *Sci. Rep.* **6**, 20423; doi: 10.1038/srep20423 (2016).

## Supplementary Material

Supplementary Information

## Figures and Tables

**Table 1:** The prediction of the variants that were identified by WES and confirmed by Sanger sequence.

Gene name	position	codons	Transcript ID	AA change	Function GVS	Region	dbSNP	ExAC database	1000 genomes	SIFT score	PolyPhen score	Grantham score	consScore GERP	Mutation Taster	CADD score
*DYNC1H1*	Chr:14,102452889	CCG > CtG	ENST00000360184	P776L	Missense	Exon CDS	Novel	Novel	Novel	0.04	1	98	5.600	Disease causing	28.1
*HSD17B7*(rs563752674)	Chr:1,162762441	C > T	ENST00000254521	None	Intron-near-splice	Intron	0.0018	0.001052	<0.01	N/A	N/A	N/A	2.350	Disease causing	16.11

Abbreviations: ExAC = Exome Aggregation Consortium database; N/A = not available.

**Table 2:** The structured elements of the variant p.P776L in *DYNC1H1* according to ACMG guidelines.

Gene	Transcript	Location	Variant	Zygosity	Classification	Disease	Parental Origin
*DYNC1H1*	NM_001376	Exon 8	c.C2327T (p.P776 L)	Heterozygous	Likely pathogenic	SMA-LED	*De novo*
