# Supplementary material for: Identification of a de novo DYNC1H1 mutation via WES according to published guidelines
Source: Sci Rep. 2016 Feb 5;6:20423. doi: 10.1038/srep20423 (PMC4742772; doi:10.1038/srep20423)
Supplement: Supplementary Information [file srep20423-s1.pdf]

A *de novo* mutation in *DYNC1H1* identified by the combination of WES and related guidelines

Dongxue Ding[1], Zhao Chen[1], Kai Li[1], Zhe Long[1], Wei Ye[1], Zhaoli Tang[1], Kun Xia[3], Rong Qiu[4], Beisha Tang[1,2,3], Hong Jiang[1,2,3*]

[1]Department of Neurology, Xiangya Hospital, Central South University, Changsha, Hunan, P.R. China

[2]Key Laboratory of Hunan Province in Neurodegenerative Disorders, Central South University, Changsha, Hunan, P.R. China

[3]State Key Laboratory of Medical Genetics, Central South University, Changsha, Hunan, P.R. China

[4]School of Information Science and Engineering, Central South University, Changsha, China

[*]Corresponding author

Phone number: +86-731-84327216

Fax number: (86)731-84327332

Post address: Department of Neurology, Xiangya Hospital, 87 of xiangya Road, Changsha, Hunan Province, China

E-mail address: jianghong73868@126.com (HJ)

Supplementary Material

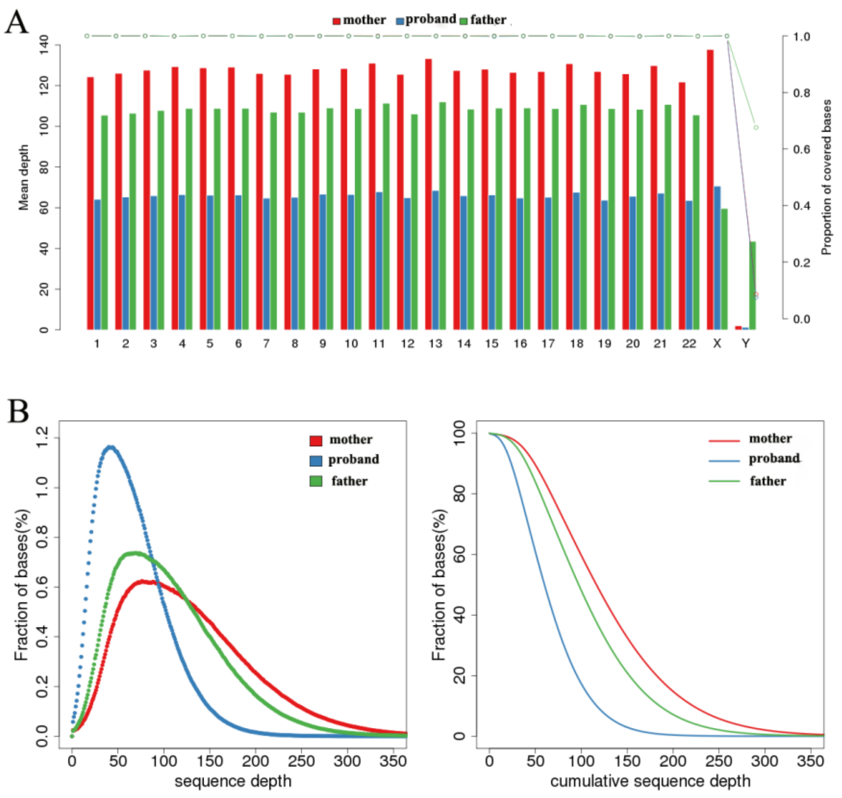

Supplementary Figure 1 Mean sequencing depth and depth distribution in target regions of the proband and her patients

SureSelect Human All ExonV5 Kit (Agilent) was used for exome capture and IlluminaHiseq 2500 platform (San Diego, CA) for sequencing of the genomic DNA of theproband and her parents. Raw data of the proband is 5.37G. While for her father and mother, the raw data are 9.52G and 11.43G, respectively. Mean sequencing depth and proportion of covered bases of each chromosome of the proband (A), the distribution of per-base sequencing depth and cumulative depthdistribution in target regions for gDNA sample of the proband (B).

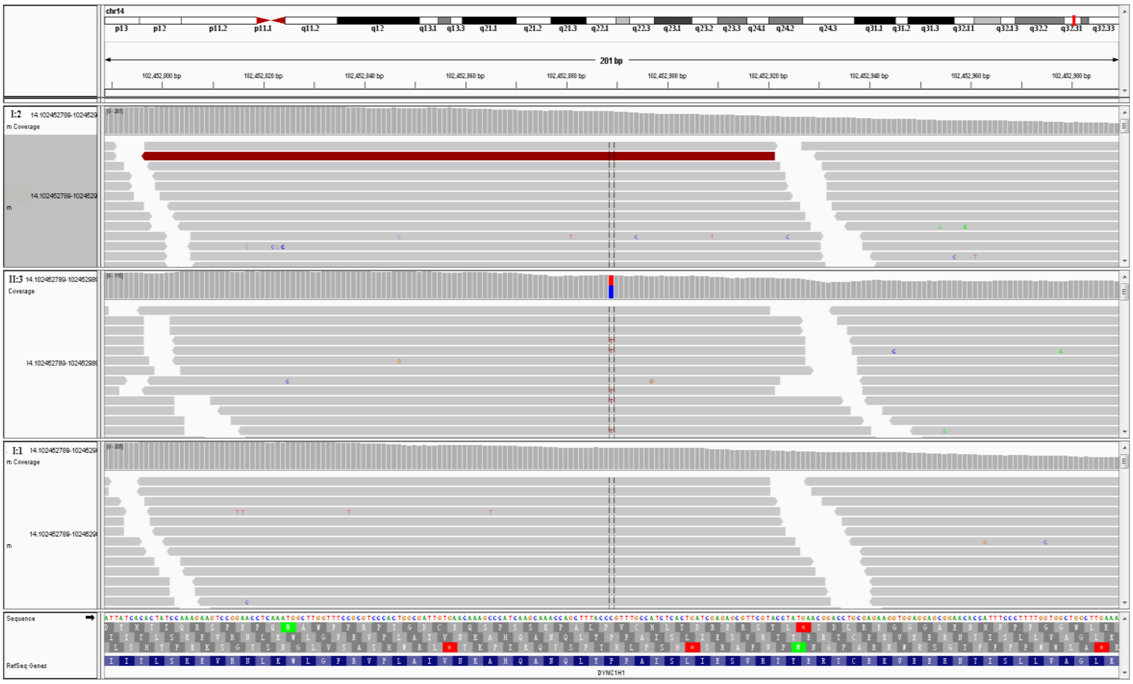

Supplementary Figure 2 IGV browser view of the *de novo*SNV(c.2327C>T, p.P776L) in *DYNC1H1*.

Top panel shows the mutation location indicated by a red box. Middle panels depict representative individual reads as well as the relative coverage per base pair for mother, probandand father. Colored coverage columns indicate the location of the SNV. Lower panel shows reference sequence and translated amino acids.

Supplementary Section 1 The pathogenic description of the variant p.P776L according to ACMG guidelines

Mutations in *DYNC1H1* have been reported previously in patients with SMA-LED [20,26,27]. The p. P776L variant in the *DYNC1H1* gene has not been published to our knowledge but was observed as a *de novo* occurrence in this pair of twins whose phenotype matches SMA-LED described for this gene. Multiple lines of computational evidence predict this variant is probably damaging to the protein structure, function, or protein-protein interaction. The p. P776L variant is a conservative amino acid substitution, which occurs at a position that is conserved across species. In addition, the predictions of *in silico* analysis tools providing a higher probability that this variant identified in our study would be pathogenic. Many missense pathogenic variants in nearby residues in the *DYNC1H1* gene have been reported in association with SMA-LED, supporting the functional importance of this region of protein. p. P776L is therefore interpreted to be likely pathogenic for SMA-LED and act in a dominant manner.

Supplementary Table S1 Results of Sanger sequencing of other genes identified by WES

Inheritance	Gene	Zygosity	Position	Ref>Alt	AA-change	Functional	Transcript ID	MAF	I:1	I:2	II:1	II:2	II:3	II:4
Recessive	*HSD17B7*	homozygous	Chr 1.162762441	C>T	N/A	splicing	ENST00000254521	0.0018 (dbSNP)	WT/ mut	WT/ mut	WT/WT	mut/mut	mut/mut	WT/WT
	MYH6	homozygous	Chr14.23858273	G>T	N/A	splicing	ENST00000356287.3	0.001936 (ExAC)	WT/mut	WT/ mut	WT/mut	mut/mut	mut/mut	mut/mut
	PCLO	homozygous	Chr 7.82581493	C>CATA	p.D2926delinsYD	Nonframeshift insertion	ENST00000423517.2	Novel	WT/mut	WT/mut	mut/mut	mut/mut	mut/mut	WT/mut
	DIXDC1	homozygous	Chr 11.111853108	C>CA	p.P272fs	Frameshift insertion	ENST00000440460	Novel	WT/WT	WT/WT	WT/WT	WT/WT	WT/WT	WT/WT
	BPTF	homozygous	Chr 17.65955779	TCCA>TCCA GCCCCACCA	p.P2684delinsPAP P	Nonframeshif tinsertion	ENST00000335221.5	Novel	WT/WT	WT/WT	WT/WT	WT/WT	WT/WT	WT/WT
Recessive	*MUC19*	heterozygous	Chr 12.40959559	T>A	Unknown	missense	ENST00000454784.4	Novel	WT/ mut	WT/WT	WT/ mut	WT/WT	WT/WT	WT/WT
	MUC19	heterozygous	Chr 12.40961397	C>T	N/A	splicing	ENST00000454784.4	Novel	WT/WT	WT/ mut	WT/ mut	WT/WT	WT/WT	WT/WT
Sporadic (De novo)	*PHOX2B*	heterozygous	Chr 4.41748010	CGC> CGCGGC	p.A253delinsAA	Nonframeshift insertion	ENST00000226382.2	Novel	WT/WT	WT/WT	WT/WT	WT/WT	WT/WT	WT/WT
	AKAP12	heterozygous	Chr 6.151674121	ATTT> AGGTTTT	N/A	Nonframeshift substitution	ENST00000253332.1	Novel	WT/WT	WT/WT	WT/WT	WT/WT	WT/WT	WT/WT
	NEK5	heterozygous	Chr 13.52701491	CTT> CTTGTT	N/A	splicing	ENST00000355568.4	Novel	WT/WT	WT/WT	WT/WT	WT/WT	WT/WT	WT/WT
	DYNC1H1	heterozygous	Chr 14 102452889	C>T	p.P776L	Missense SNV	ENST00000360184	Novel	WT/WT	WT/WT	WT/WT	WT/ mut	WT/ mut	WT/WT

Abbreviations: N/A=not available; WT= wild type; mut= mutation

Supplementary Table S2 Clinical and genetic features of patients with *DYNC1H1* mutations

Domain	Mutations	Inheritance	Presentation	Motor ability	Muscle weakness and atrophy	DTR	Cognitive impairment	Other	Reference
MD	p.H3822P	*De novo*	MCD	Delayed motor milestones	N/A	Reduced	Mild	N/A	Ng SB et al. [8]
SD	p.E1518K	*De novo*	MCD	Never walk,spastic tetraplegia,	N/A	N/A	Severe	Congenitalclubfeet, generalised epileptic seizures, small hands and feet, kyphoscoliosis, progressive swallowing difficulties	Willemsen MH et al.. [20]
MD	p.R3384Q	*De novo*	MCD	Bedridden, spastic tetraplegia	N/A	N/A	Severe	Microcephaly, early onset epilepsy, foot deformities, pachygyria	Poirier K et al. [17]
MD	p.R3344Q	*De novo*	MCD	N/A	N/A	N/A	Severe	Autistic features, epilepsy, post. Agyria, Nodular Heterotopia	
MD	p.K3336N	*De novo*	MCD	Bedridden, spastic tetraplegia	N/A	N/A	Severe	Microcephaly, early onset epilepsy, Foot deformities	
MD	p.K3241T	*Familial*	MCD	N/A	N/A	N/A	Mild	Focal seizures, Pachygyria	
MD	p.R1962C	*De novo*	MCD	N/A	N/A	N/A	Severe	Focal seizures, Pachygyria	
SD	p.R1567Q	*De novo*	MCD	N/A	N/A	N/A	Severe	Foot defonities, polymicrogyria	
SD	p.del659-662	*De novo*	MCD	Bedridden, spastic tetraplegia	N/A	N/A	Severe	Early onset epilepsy, Microcephaly, Pachygyria	
SD	p.K129I	*De novo*	MCD	N/A	N/A	N/A	Severe	Late onset epilepsy, Pachygyria	
MD	p.E2616K	AD heterozygous	SMA-LED and LD	Waddling gait, falls,	LL P>D, LL dist	Reduced in LL	Mild	N/A	Scoto M et al. [25]
SD	p.R1603T	*De novo*	MCD and SMA-LED	Talipes and leg Contractures,	LL P>D, LL dist	Absent in LL	Neurodevelopmental delay	Epilepsy, exotropia, valgus feet	
SD	p.Y970C	AD heterozygous	MCD and SMA-LED	Delayed motor milestones, waddling gait	LL P>D, LL D>P	Reduced in LL	Mild	N/A	
SD	p.W673C	AD heterozygous	SMA-LED	Waddling gait	LL	Absent in LL	No	CHD, talipes	
SD	p.V612M	AD heterozygous	SMA-LED	N/A	LL	Reduced in LL	No	Talipes,CHD	

SD	p.E603V	AD heterozygous	SMA-LED	N/A	LL	Reduced in LL	No	N/A	
SD	p.R598L	AD heterozygous	SMA-LED	N/A	LL dist;<br>LL P>D	Absent in knee;<br>Reduce in Achilles	No	Equinovarus feet	
SD	p.R598C	AD heterozygous	SMA-LED	Waddling gait,	LL D>P;<br>LL P>D	Reduced in LL	No	Valgus feet, Talipes	
SD	p.I584L	AD heterozygous	SMA-LED	N/A	LL P>D	Reduced in LL	Mild	Exotropia	
SD	p.M581L	Heterozygous	SMA-LED and LD	Waddling gait,	LL D>P; LL Prox	Reduced in LL	No	Valgus feet, Talipes	
SD	p.R399G exon8	Codominant homozygous	MCD and SMA-LED	N/A	LL	Reduced in LL	Moderate	Valgus feet	
SD	p.D338N	Heterozygou	SMA-LED	N/A	LL>UL;<br>UL>LL dist	N/A	Speech delay	Talipes, arthrogryposis<br>LL>UL	
SD	p.R264G	*De novo*	MCD and SMA-LED	P>D	D>P of LL	Reduced in LL	N/A	Valgus feet	
SD	p.H306R	*Familiar*	SMA-LED	Moderate proximal lower, waddling gait	LL Prox	Decreased	No	Gower's sign, waddling gait	Tsurusaki Y et al. [24]
SD	p.H306R	Heterozygous	CMT2O(HMSN)	Distal lower limb (one of the patients)	LL dist	Normal	N/A	*Pescavus*; reduced proprioception, pin-prick, significant neuropathic pain; depression and paraphrenia; extrapyramidal features probably due to antipsychotics	Weedon et al. [15]
SD	p.R598C	Heterozygous	CMT2 (Axonal HMSN)	Delayed motor milestones, waddling gait, upper limb involved in the later stage	LL	Reduced /absent	N/A	Pescavus, mild sensory abnormalities, mild scoliosis of the thoracic spine	Peeters K et al. [21]
SD	p.R264L	*De novo*	SMA	Delayed motor milestones, never walking	LL	Absent	No	Contractures of all lower limb joints, fracture of the right femur and luxation of the right hip joint, muscle hypotony, mild	

				independently				ventricular enlargement, scoliosis of the spine	
ND (1150-1300)	p.Q1194R (exon 16)	*De novo*	SMA and MCD	Waddling gait	LL	N/A	Mild	*Pescavus*, congenital foot deformity , right curve scoliosis	Fiorillo C et al. [23]
MD	p.E3048K (exon 47)	*De novo*	MCD and SMA-LED	Delayed motor milestones;	LL P>D, mild weakness in shoulder girdle muscles	Globally absent	Mild	Foot deformity; cortical malformations	
SD	p.I584L	AD heterozygous	SMA-LED	N/A	LL P>D	Reduced in knee reflexion	No	Mild pescavus, hip abduction	Harms MB et al. [18]
SD	p.K671E	AD heterozygous	SMA-LED	Waddling gait	LL P>D	Reduced in knee reflexes	No	Lumbar lordosis	
SD	p.Y970C	AD heterozygous	SMA-LED	Significant motor delay	N/A	N/A	Mild	N/A	
SD	p.R598C	*De novo*	SMA-LED	Delayed walking, waddling gait	LLdist	Absent in LL	No	congenital scoliosis, equinovarus, and L5/S1 left hemivertebra, febrile seizures,	Punetha J et al. [22]
SD	p.R598C	*De novo*	SMA-LED	Delayed motor milestones, waddling gait	LL	Reduced	No	Positive Gower’s sign, pescavus of the rightfoot	Strickland AV et al. [16]
SD	p.D1062G	*De novo*	cHSP	Lower-limb predominant spastic tetraparesis	N/A	N/A	Cognitive deficits	Behavioral disturbances, mild ataxia, treatment-refractory focal epilepsy	
MD	p.S3360G	AD heterozygous	SMA-LED	Delayed motor milestones, gait abnormalities, and frequent falls	LL dist	Absent in the LL	N/A	Foot deformity at birth, *pescavus*, lumbar hyperlordosis	

Abbreviations: MD=motor domain;SD=stem domain; ND=neck domain; DTR=deep tendon reflexes; LL=lower limb; prox=proximal; dist=distal; N/A=not available; SMA-LED=spinal muscular atrophy with lower extremity predominance; MCD=malformations of cortical development; c HSP=complex hereditary spastic paraplegia;UL=upper limbs.
